# Research progress on the regulation of microglia and astrocyte functions by protein lactylation in cerebral ischemia-reperfusion injury: A review

**DOI:** 10.1097/MD.0000000000048297

**Published:** 2026-04-17

**Authors:** Hang-hang Song, Xi-cheng Jiang

**Affiliations:** a School of Basic Medical Sciences, Heilongjiang University of Traditional Chinese Medicine, Harbin, China.

**Keywords:** astrocytes, cerebral ischemia-reperfusion injury, microglia, protein lactation

## Abstract

Protein lactylation, a novel posttranslational modification, has garnered significant attention for its regulatory mechanisms in cerebral ischemia-reperfusion injury. This review highlights the critical roles of protein lactylation in regulating microglia and astrocytes functions. By modulating the activation of microglia and astrocytes, inflammatory responses, and antioxidative stress, protein lactylation plays a pivotal role in neural repair and regeneration. Following cerebral ischemia-reperfusion injury, lactylation enhances the phagocytic activity of microglia, facilitates the clearance of necrotic cells, reduces the secretion of pro-inflammatory cytokines, and thus protects neurons. In astrocytes, lactylation modification supports blood–brain barrier integrity, provides nutritional support, and regulates antioxidative enzymes, further promoting neuronal survival and functional recovery. Further investigation into the molecular mechanisms of protein lactylation, its specific roles in glial cells, the development of novel lactylation regulators, and its interactions with other posttranslational modifications will offer new insights and directions for the treatment of cerebral ischemia-reperfusion injury.

## 1. Introduction

**Figure 1.**
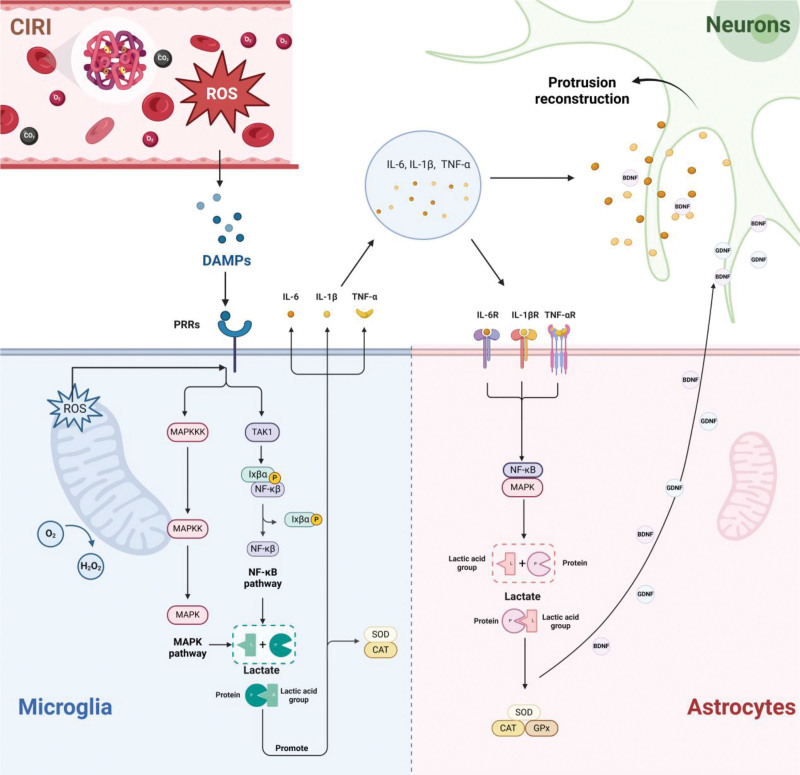
The mechanism of glial cell function involves multiple key signaling and molecular events. Reactive oxygen species (ROS) production and the release of damage-associated molecular patterns (DAMPs) activate microglia through the NF-κB and MAPK signaling pathways, leading to increased expression of pro-inflammatory factors such as IL-1β, TNF-α, and IL-6. Protein lactylation modulates these pathways, reducing the expression of pro-inflammatory factors and enhancing the anti-inflammatory response. Meanwhile, astrocytes detect inflammatory factors, including IL-1β and TNF-α released by microglia, through their receptors, which in turn activate the NF-κB and MAPK signaling pathways. This activation enhances anti-inflammatory responses and increases antioxidant capacity. Additionally, it regulates the proliferation and secretion of neurotrophic factors, ultimately promoting neuronal survival, axonal extension, and synaptic reconstruction. BDNF = brain-derived neurotrophic factor, CAT = catalase, CIRI = cerebral ischemia–reperfusion injury, DAMPs = damage-associated molecular patterns, GDNF = glial cell line-derived neurotrophic factor, GPx = glutathione peroxidase, IL = interleukin, MAPK = mitogen-activated protein kinase, NF-κB = nuclear factor kappa-light-chain-enhancer of activated B cells, NGF = nerve growth factor, PRR = pattern recognition receptor, ROS = reactive oxygen species, SOD = superoxide dismutase, TAK1 = transforming growth factor-β-activated kinase 1, TNF = tumor necrosis factor.

Cerebral ischemia-reperfusion injury is a common phenomenon in ischemic stroke and one of the major causes of high morbidity and disability. During reperfusion, the restoration of blood flow triggers oxidative stress, inflammatory responses, and cell death, significantly exacerbating brain damage and adversely affecting patient outcomes.^[1]^ Glial cells, including microglia and astrocytes, play critical roles in cerebral ischemia-reperfusion injury. Microglia, as the primary immune cells of the central nervous system, are rapidly activated by injury signals and release large amounts of inflammatory cytokines while clearing necrotic cells. However, excessive activation of microglia aggravates inflammatory responses and exacerbates tissue damage.^[2]^ Astrocytes, on the other hand, maintain blood–brain barrier integrity, provide nutritional support, and regulate ion balance to ensure neuronal function. Post-injury, astrocytes also participate in neural repair and regeneration.^[3]^ Protein lactylation, a novel posttranslational modification, involves the covalent attachment of lactate groups to proteins, affecting their function and stability. It plays an essential role in cellular metabolism and signal transduction by modulating key enzymes and signaling pathways.^[4]^ Recent studies suggest that protein lactylation is critically involved in the nervous system, particularly in neuroprotection, inflammation regulation, and cell survival. This review aims to summarize the role of protein lactylation in cerebral ischemia-reperfusion injury, focusing on its regulatory mechanisms in microglia and astrocytes function. By highlighting the latest research advances, we seek to provide a theoretical basis and scientific support for developing novel therapeutic strategies for cerebral ischemia-reperfusion injury (Fig. 1).

## 2. Microglia and astrocytes in cerebral ischemia-reperfusion injury

Microglia and astrocytes, 2 key types of glial cells in the central nervous system, play dual roles in cerebral ischemia-reperfusioninjury. While they can protect neurons and promote tissue repair, their excessive activation may exacerbate inflammatory responses, leading to secondary damage. Microglia, the primary immune cells in the central nervous system, are responsible for immune surveillance, phagocytosis of necrotic cells, and regulation of inflammatory responses. Under normal conditions, microglia remain in a resting state, constantly scanning their surroundings for pathological signals.^[5]^ However, during cerebral ischemia-reperfusion injury, microglia are rapidly activated. Through pathways such as Toll-like receptors, nuclear factor kappa-light-chain-enhancer of activated B cells (NF-κB), and mitogen-activated protein kinase, microglia transition from a resting to an activated state, characterized by an enlarged cell body and retracted processes. Activated microglia secrete large amounts of pro-inflammatory cytokines, including interleukin (IL)-1β, IL-6, and tumor necrosis factor (TNF)-α, amplifying the local inflammatory response. Additionally, activated microglia possess robust phagocytic capabilities, clearing necrotic or apoptotic neurons and cellular debris to limit damage and promote repair.^[6,7]^ However, excessive microglial activation can generate reactive oxygen species (ROS) and nitric oxide, exacerbating oxidative stress, damaging neurons and the blood–brain barrier, and leading to secondary injury. Therefore, microglia exhibit dual roles in brain injury: they can be protective by promoting repair but may also contribute to neurotoxicity through excessive inflammation. Astrocytes also play critical roles in cerebral ischemia-reperfusion injury. Their essential functions include maintaining blood–brain barrier integrity, providing nutritional support, and regulating ionic balance around neurons.^[8]^ Following injury, astrocytes rapidly activate, characterized by an enlarged cell body and shorter, thicker processes, a phenomenon known as reactive astrogliosis or glial scar formation. Activated astrocytes mitigate local inflammation by secreting anti-inflammatory factors and antioxidative enzymes. They also participate in matrix remodeling through the secretion of matrix metalloproteinases, promoting tissue repair. Moreover, astrocytes enhance neuronal survival and functional recovery by secreting neurotrophic factors.^[9,10]^ By regulating blood–brain barrier permeability, astrocytes prevent excessive infiltration of inflammatory cells, maintaining the stability of the neural microenvironment. However, excessive activation of astrocytes can lead to glial scar formation, which impedes axonal regeneration. In summary, both microglia and astrocytes exhibit protective and detrimental roles in cerebral ischemia-reperfusion injury. While they contribute to repair and protection, their overactivation may aggravate neuroinflammation and exacerbate injury.^[11]^ Understanding the activation mechanisms of these glial cells and their roles in the inflammatory response will aid in developing targeted therapeutic strategies to mitigate neuroinflammation and promote functional recovery.

## 3. Cerebral ischemia-reperfusion injury and protein lactic acidification

### 3.1. Molecular mechanisms of protein lactylation

Protein lactylation is a novel posttranslational modification involving the covalent attachment of lactyl groups to lysine residues on proteins, altering their function and structure. This process is primarily mediated by enzymes such as lactate dehydrogenase (LDH), which not only converts pyruvate to lactate during glycolysis but also catalyzes the attachment of lactyl groups to protein lysine residues, forming lactylation modifications.^[12]^ This dynamic process participates in various physiological and pathological processes within cells. Protein lactylation modifies the 3-dimensional structure of proteins, influencing their functionality and stability. It plays a pivotal role in regulating cellular functions and signal transduction by modifying key signaling molecules in pathways such as PI3K/Akt and NF-κB. The PI3K/Akt pathway is a major regulator of cell survival, proliferation, and metabolism. Lactylation modulates the activity of PI3K and Akt proteins, affecting downstream targets such as mTOR and GSK-3β, thereby regulating cell growth and survival.^[13,14]^ In the NF-κB pathway, which governs inflammation and immune responses, lactylation modifies regulatory proteins like IκBα and p65. This modification stabilizes IκBα, preventing its degradation, which in turn inhibits NF-κB activation and reduces inflammation.^[15]^ Furthermore, lactylation interacts with other posttranslational modifications, such as acetylation and phosphorylation, creating a complex regulatory network that finely tunes cellular functions. For example, research by Yang et al^[16]^ demonstrated that lactylation competes with acetylation at lysine residues, influencing acetylation-mediated gene expression and metabolic regulation. Similarly, interactions between lactylation and phosphorylation can alter the charge and conformation of proteins, affecting the dynamic balance and response speed of signaling pathways. In summary, protein lactylation, as a critical posttranslational modification, regulates protein functionality and structure, significantly impacting pathways like PI3K/Akt and NF-κB. Understanding the molecular mechanisms of lactylation sheds light on its vital roles in cellular physiology and pathology, offering new therapeutic strategies for cerebral ischemia-reperfusion injury.

### 3.2. Specific regulation of protein lactylation in microglia and astrocytes

Protein lactylation, as a novel posttranslational modification, significantly impacts the activation and function of glial cells, particularly microglia and astrocytes. These glial cells play pivotal roles in neuroinflammation and tissue repair within the central nervous system (CNS). In microglia, the principal immune cells of the CNS, protein lactylation regulates their activation states and immune functions. Lactylation modifies key proteins within microglia, influencing their signaling pathways.^[17]^ Specifically, lactylation can modulate the NF-κB signaling pathway, reducing pro-inflammatory responses. This mechanism involves stabilizing the IκBα protein, inhibiting NF-κB nuclear translocation, and subsequently decreasing the expression of pro-inflammatory cytokines such as IL-1β, TNF-α, and IL-6. By mitigating excessive inflammation, lactylation protects neurons from the detrimental effects of inflammatory mediators. Additionally, lactylation can regulate microglial migration and phagocytosis, enhancing their capacity to clear necrotic cells and promote tissue repair in damaged regions.^[18]^ In astrocytes, lactylation plays a crucial role in modulating their responses and functions. Astrocytes are essential for maintaining the blood–brain barrier, providing nutritional support, and regulating ionic balance.^[19]^ Upon ischemia-reperfusion injury, astrocytes rapidly activate, undergoing morphological changes characterized by increased cell body size and hypertrophied processes. Lactylation influences astrocytic proliferation and functionality by modulating key signaling pathways such as PI3K/Akt. Zhang et al^[20]^ demonstrated that lactylation enhances the PI3K/Akt signaling pathway, promoting astrocyte survival and proliferation and increasing the secretion of neurotrophic factors. These factors support neuronal survival and regeneration. Furthermore, Jain et al^[21]^ reported that lactylation can regulate the JAK/STAT pathway in astrocytes, influencing their reactive gliosis and extracellular matrix remodeling. This modulation facilitates repair at the site of injury and supports neural regeneration. In summary, protein lactylation regulates microglial inflammation and phagocytic capabilities, reducing excessive inflammation and protecting neurons. Concurrently, it modulates astrocytic proliferation and neurotrophic factor secretion, enhancing neural tissue repair and regeneration. These findings underscore the potential of targeting protein lactylation as a therapeutic approach for CNS disorders involving ischemia-reperfusion injury.

## 4. Regulation of protein lactylation on microglia and astrocytes function

### 4.1. Regulation of neuroinflammation by lactylation

Protein lactylation plays a pivotal role in regulating neuroinflammation, particularly in microglia and astrocytes. Microglia, the primary immune cells of the CNS, act as first responders during neuroinflammatory processes. Lactylation modulates microglial activation and functionality by influencing key signaling pathways. Specifically, lactylation inhibits the expression of pro-inflammatory cytokines, such as IL-1β, TNF-α, and IL-6, through the regulation of NF-κB and mitogen-activated protein kinase pathways.^[22]^ This suppression prevents excessive inflammatory responses, thereby protecting neurons from further damage. Additionally, protein lactylation enhances microglial migration and phagocytic capabilities, facilitating the clearance of cellular debris and pathogens. By modulating these functions, lactylation plays a critical role in controlling the spread and exacerbation of neuroinflammation. Furthermore, lactylation not only inhibits pro-inflammatory responses in microglia but also promotes their polarization toward an anti-inflammatory phenotype, thereby increasing the secretion of anti-inflammatory cytokines and balancing the inflammatory response.^[23]^ In astrocytes, protein lactylation also exerts significant regulatory effects. Astrocytes are crucial for maintaining the integrity of the blood–brain barrier and modulating the neuronal microenvironment. Upon CNS injury, astrocytes become activated, characterized by increased cell body size and process hypertrophy. Lactylation modulates key signaling pathways, such as PI3K/Akt, within astrocytes, thereby influencing their proliferation and secretory functions. This modification enhances astrocytic anti-inflammatory responses, increasing the secretion of anti-inflammatory cytokines to mitigate inflammation and protect neurons.^[24]^ Additionally, lactylation augments the antioxidative capacity of astrocytes, enabling them to better combat oxidative stress and reduce oxidative damage. The role of lactylation in neuroinflammation extends beyond individual cell types. By regulating the interplay between microglia and astrocytes, lactylation influences the overall progression of inflammation. These 2 glial cell types communicate via various cytokines and mediators, collectively maintaining CNS homeostasis. Lactylation fine-tunes these intercellular signaling pathways, coordinating inflammatory responses and fostering neuroprotection and repair.^[25]^ In summary, protein lactylation significantly impacts the progression of neuroinflammation by modulating the activation of microglia and astrocytes. Through balancing pro-inflammatory and anti-inflammatory responses and enhancing antioxidative capacities, lactylation contributes to neuronal protection and tissue repair, highlighting its potential as a therapeutic target for neuroinflammatory disorders.

### 4.2. Effects of lactylation on oxidative stress resistance

Protein lactylation plays a pivotal role in regulating oxidative stress responses, particularly through mechanisms in glial cells. It influences cellular resistance to oxidative stress by modulating the activity of key antioxidant enzymes. Superoxide dismutase (SOD) and catalase (CAT) are 2 major antioxidant enzymes responsible for scavenging ROS. Lactylation modifies these enzymes, altering their activity and stability, thereby enhancing their antioxidant functions.^[26,27]^ In microglia, protein lactylation enhances antioxidant capacity by modifying critical antioxidant enzymes, such as SOD and CAT. This modification increases their activity, enabling microglia to more effectively clear ROS and mitigate oxidative stress-induced damage. This regulation not only protects microglia from oxidative injury but also reduces ROS accumulation, safeguarding surrounding neurons and other cells. Additionally, lactylation can regulate the nuclear factor erythroid 2-related factor 2 (Nrf2) pathway, a crucial transcription factor that induces the expression of antioxidant enzymes. Research by Pourbagher-Shahri et al^[28]^ demonstrates that lactylation may stabilize Nrf2, promoting its nuclear translocation and transcriptional activity, thereby enhancing the expression of antioxidant genes. In astrocytes, protein lactylation also significantly impacts oxidative stress resistance. Astrocytes play a critical role in maintaining neuronal microenvironment stability and protecting neurons from oxidative damage. Lactylation enhances astrocytic antioxidant enzyme activity, boosting their ROS-scavenging capability and reducing oxidative stress-induced cellular damage.^[29]^ Specifically, lactylation increases the activity of SOD and CAT, enabling astrocytes to neutralize ROS more efficiently and protect neurons. Furthermore, lactylation regulates the activity of glutathione peroxidase (GPx), another key antioxidant enzyme, which reduces hydrogen peroxide and organic hydroperoxides to water and alcohol. Pei et al^[30]^ reported that lactylation enhances GPx functionality, strengthening cellular defenses against oxidative stress. Beyond these enzymatic modifications, lactylation influences additional signaling pathways to bolster oxidative stress resistance. It enhances cell survival and antioxidant capacity through the PI3K/Akt pathway. The PI3K/Akt pathway regulates cellular proliferation, survival, and metabolism, and lactylation activates this pathway to increase antioxidant enzyme expression and activity, thereby improving oxidative defense.^[31]^ Zhou et al^[32]^ demonstrated that lactylation also regulates the AMP-activated protein kinase pathway, which promotes energy metabolism and oxidative stress responses. As an energy-sensing kinase, AMPK activation via lactylation improves cellular resilience against oxidative stress. In summary, protein lactylation plays a vital role in counteracting oxidative stress by modulating the activity of antioxidant enzymes and signaling pathways in glial cells. By enhancing the activity of enzymes such as SOD, CAT, and GPx, lactylation boosts the oxidative stress resistance of microglia and astrocytes, mitigating cellular damage. This regulatory mechanism not only protects glial cells but also reduces ROS accumulation, thereby safeguarding neurons and other cells and maintaining the stability and functionality of the nervous system.

### 4.3. Role of lactylation in neural repair and regeneration

Protein lactylation plays a crucial role in regulating glial cells, particularly in facilitating neural repair and regeneration. Glial cells, including microglia and astrocytes, are central to maintaining homeostasis, supporting neuronal functions, and participating in the repair processes of the nervous system. Following neural injury, lactylation regulates glial cell functions through various mechanisms, promoting neural repair and regeneration. In microglia, protein lactylation modulates their activation and secretory functions, enhancing their role in the repair process. Upon receiving injury signals, microglia become rapidly activated and migrate to the damaged site, where they phagocytose cellular debris and secrete neurotrophic factors to promote neural repair.^[33]^ Lactylation enhances the phagocytic ability of microglia, aiding in the clearance of debris and pathogens at the injury site, thereby reducing inflammatory responses. Furthermore, lactylation regulates the secretory profile of microglia by increasing the release of anti-inflammatory cytokines such as IL-10 and TGF-β, minimizing inflammation-induced damage to surrounding neurons. Through these mechanisms, lactylation significantly contributes to maintaining homeostasis in the nervous system during neural repair.^[34,35]^ Astrocytes play a pivotal role in neural repair and regeneration, and lactylation promotes this process by regulating their proliferation and secretory functions. Lactylation enhances astrocyte survival and proliferation, enabling them to rapidly cover damaged areas and form protective barriers to prevent further injury.^[36]^ Morrison et al^[37]^ demonstrated that lactylation regulates the secretion of brain-derived neurotrophic factor and glial-derived neurotrophic factor by astrocytes. These neurotrophic factors support neuronal survival and regeneration, facilitating axonal extension and synaptic remodeling. Additionally, lactylation is indispensable in modulating astrocytes’ extracellular matrix remodeling capabilities. It increases the secretion of matrix metalloproteinases, facilitating extracellular matrix degradation and reconstruction, thereby creating a conducive microenvironment for neuronal regeneration.^[38,39]^ Lactylation also enhances astrocytic antioxidant enzyme activity, bolstering their capacity to counter oxidative stress and mitigating oxidative damage to neurons, which supports neural repair.^[40]^ Kiaie et al^[41]^ reported that lactylation’s regulatory effects extend beyond acute neural injury repair and hold potential therapeutic value in chronic neurodegenerative diseases. By modulating lactylation, glial cell repair functions can be enhanced, alleviating the long-term effects of chronic inflammation and oxidative stress on neurons, thus promoting neural regeneration and functional recovery. In summary, protein lactylation facilitates neural repair and regeneration by regulating the phagocytic and anti-inflammatory functions of microglia and the proliferative, neurotrophic factor secretion, and extracellular matrix remodeling capabilities of astrocytes. These mechanisms not only play a vital role in brain ischemia-reperfusion injury but also have therapeutic potential for various acute and chronic neurodegenerative diseases.

## 5. Application of protein lactylation as a therapeutic target

### 5.1. Therapeutic strategies targeting protein lactylation

Targeting protein lactylation offers promising therapeutic potential in the study of neurological disorders. Current research focuses on developing small-molecule inhibitors and activators to regulate protein lactylation levels, influencing cellular functions through key enzymes such as LDH.^[42]^ LDH inhibitors reduce lactate production, thereby lowering protein lactylation levels and mitigating inflammation and cellular damage caused by excessive lactylation. Wu et al^[43]^ demonstrated that small-molecule activators, which enhance LDH activity or facilitate lactate group attachment, can modulate protein lactylation levels, improve cellular metabolism and function, and show potential therapeutic benefits. CRISPR/Cas9 gene editing technology also presents significant applications, enabling precise manipulation of genes related to lactylation. By knocking out or activating LDH genes, researchers can directly modulate lactylation levels to study their roles under various pathological conditions. This technology not only supports foundational research but also opens new pathways for developing gene therapy approaches. Additional strategies for targeting protein lactylation include the use of specific antibodies and gene silencing techniques to regulate the activity of enzymes involved in lactylation.^[44]^ For instance, designing specific antibodies against LDH or other lactylation-related enzymes can block their function, allowing precise modulation of lactylation levels. Gene silencing technologies, such as siRNA and shRNA, suppress the expression of lactylation-related genes, reducing protein lactylation and achieving therapeutic goals. Therapeutic approaches targeting protein lactylation have demonstrated remarkable effects in various disease models, particularly in neurological disorders. By regulating protein lactylation, it is possible to alleviate neuroinflammation, protect neurons, and promote neural repair and regeneration.^[45]^ With continued research and technological advances, more efficient and safe lactylation modulators are expected to be developed and applied clinically. In summary, therapeutic strategies targeting protein lactylation ranging from small-molecule inhibitors and activators to gene editing technologies show immense potential for modulating lactylation levels in neurological diseases. These methods effectively regulate protein lactylation, reducing inflammation and cellular damage, and fostering neural repair and regeneration. They represent a novel direction and bring new hope for treating related disorders.

### 5.2. Multi-target combination therapy regulating protein lactylation

Combining anti-inflammatory drugs with protein lactylation regulators offers an effective strategy to reduce inflammation and protect neurons. Nonsteroidal anti-inflammatory drugs and corticosteroids alleviate neuroinflammation by inhibiting the production and release of inflammatory mediators. When used in combination, protein lactylation regulators modulate the activity of key enzymes like LDH, reducing protein lactylation levels, thereby suppressing pro-inflammatory cytokines and enhancing anti-inflammatory effects.^[46]^ Khan et al^[47]^ demonstrated that LDH inhibitors reduce lactate production and protein lactylation, mitigating inflammation and cellular damage. The combination of antioxidants and protein lactylation regulators has also shown significant efficacy. Antioxidants such as vitamin E and *N*-acetylcysteine reduce oxidative stress by scavenging ROS, while protein lactylation regulators enhance the activity of antioxidant enzymes like SOD and CAT.^[48]^ LDH inhibitors, in addition to lowering lactate production and lactylation levels, indirectly increase antioxidant enzyme activity, further amplifying the antioxidative effect. This combination strategy has proven effective in neurological disease models, simultaneously reducing inflammation and oxidative stress, protecting neurons, and promoting neural repair and regeneration. The design and application of multifunctional nanocarriers for combination therapies hold immense potential. These carriers can simultaneously deliver anti-inflammatorydrugs, antioxidants, and protein lactylation regulators to diseased areas, increasing drug concentration and therapeutic efficacy.^[49]^ Studies by Zielińska et al^[50]^ and Guimarães et al^[51]^ highlight the use of liposomes and polymeric nanoparticles to encapsulate multiple drugs within a single carrier. By modifying these carriers with specific targeting molecules, they achieve precise delivery to sites of neural injury. Nanocarriers not only provide efficient delivery but also enable controlled drug release, offering sustained therapeutic effects while reducing dosage frequency and side effects. Moreover, by incorporating protective molecules like polyethylene glycol, nanotechnology improves the bioavailability and stability of drugs, enhancing circulation time and targeting efficiency.^[52]^ Beyond drug delivery, multifunctional nanocarriers can integrate imaging and diagnostic functions, enabling theranostics combining therapy and monitoring into a single system. This dual functionality is particularly significant for neurological disorders, where tracking treatment progress is critical. In summary, multi-target combination therapy regulating protein lactylation, through the integration of anti-inflammatory drugs, antioxidants, and advanced nanocarrier systems, exhibits considerable therapeutic potential (Table 1).^[53]^ By addressing multiple pathological processes reducing inflammation and oxidative stress, protecting neurons, and promoting neural repair and regeneration this approach provides innovative insights and directions for the treatment of ischemia-reperfusion brain injury.

**Table 1:** Summary of applications of protein lactation-related targets.

Applications of protein lactation-related targets	Mechanism of action	References
LDH inhibitors	Interfering with LDH key enzyme activity	^[42,43]^
Gene editing	CRISPR/Cas9 gene editing technology, knockout or activation of LDH gene	^[53]^
Targeted inhibition of protein lactylation		
Specific antibodies	Blocks enzymes involved in protein lactylation	^[44]^
Gene silencing	Interfering with the expression of RNA involved in protein lactylation	^[44]^
Drug combination application		
NSAIDs + glucocorticoids + LDH inhibitors	LDH inhibitors act synergistically to inhibit inflammatory cytokine production	^[46,47]^
Vitamin E/*N*-acetylcysteine + protein lactation regulator	Antioxidants scavenge ROS, and protein lactation regulators regulate antioxidant enzyme activity	^[48]^
Nanocarriers	It can realize functions such as drug delivery, image development, and regular monitoring	^[49-52]^

LDH = lactate dehydrogenase, NSAIDs = nonsteroidal anti-inflammatory drugs, RNA = ribonucleic acid, ROS = reactive oxygen species.

## 6. Summary and outlook

Protein lactylation has been increasingly recognized as a critical player in ischemia-reperfusion brain injury. Emerging evidence suggests that protein lactylation significantly influences neuroglial cell activation, inflammatory responses, and oxidative stress, thereby contributing to neural repair and regeneration. Recent studies have demonstrated that protein lactylation alleviates neural damage by enhancing the activity of antioxidant enzymes and regulating the expression of inflammatory cytokines in microglia and astrocytes. Elucidating the molecular mechanisms and specific roles of protein emulsification in microglia and astrocytes will facilitate the development of novel protein emulsification regulators with potential clinical applications. Furthermore, investigating the interplay between protein lactylation and other posttranslational modifications, as well as their integrated regulatory networks, will provide deeper insights into its complex role in cellular function. Evaluating the therapeutic potential of protein lactylation modulation in ischemia-reperfusion brain injury could lay a foundation for innovative treatment strategies. With ongoing advancements in science and technology, protein lactylation modulators are expected to play an increasingly significant role in clinical therapy, offering promising prospects for the treatment of ischemia-reperfusion brain injury and related neurological disorders.

## Acknowledgments

We would like to thank the China Natural Science Foundation, the Heilongjiang Provincial Natural Science Foundation, and Heilongjiang University of Traditional Chinese Medicine for supporting this research.

## Author contributions

**Conceptualization:** Hang-hang Song.

**Data curation:** Hang-hang Song.

**Funding acquisition:** Xi-cheng Jiang.

**Methodology:** Xi-cheng Jiang.

**Project administration:** Xi-cheng Jiang.

**Software:** Hang-hang Song.

**Supervision:** Hang-hang Song.

**Validation:** Hang-hang Song.
